# Caregivers’ views on childcare staff’s role in promoting children’s oral health

**DOI:** 10.1186/s12903-025-05946-y

**Published:** 2025-05-24

**Authors:** Dalil Alshammari, Nia Coupe, Sarah Peters, Joanna Goldthorpe

**Affiliations:** 1 School of Health Sciences, Division of Psychology & Mental Health, University of Manchester, Manchester, United Kingdom; 2 College of Dentistry, Preventive Dentistry Department, University of Ha’il, Ha’il, Saudi Arabia; 3 School of Medical Sciences, Division of Medical Education, University of Manchester, Manchester, United Kingdom

## Abstract

**Background:**

The primary reason children in the UK are referred to hospitals for a general anaesthetic is to have decayed teeth removed. Tooth decay is a non-communicable disease which is preventable through healthy behaviours such as brushing at least twice a day with fluoride toothpaste, reducing the frequency of sugar consumption throughout the day and regular dental check-ups. Recently, oral health became a mandatory component of the England Early Years Settings’ (EYS) framework. Successful implementation of this public health strategy necessitates involvement from parents and carers, however their views currently remain unexplored. Therefore, we aim to investigate parents’ views about the role of EYS and childcare providers in promoting and discussing children’s oral health behaviours.

**Method:**

Semi-structured interviews were conducted in English and Arabic with 14 parents (11 mothers, 3 fathers) of at least one child under five years who was enrolled in any EYS (nurseries, preschools, Sure Start centres) in England. Participants were recruited through EYS settings and using online social media. Data were analysed using an inductive and latent reflexive thematic analysis.

**Results:**

The analysis was organized into three overarching themes: 1)Opportunities for parent-staff communication in early years settings are lacking. 2)Early years settings not currently considered a place for oral health. 3) Looking forward; early years settings as a partner to support children’s oral health.

**Conclusion:**

Parents have limited awareness of what oral health activities occur in EYS. They acknowledge that EYS staff can influence children’s oral health behaviours, yet also report communication barriers with EYS staff. Early years communication is primarily uni-directional, which shapes parents’ views about EYS staff’s responsibility and credibility to support their children’s oral health. To overcome these barriers, staff need training and support to effectively engage parents.

**Supplementary Information:**

The online version contains supplementary material available at 10.1186/s12903-025-05946-y.

## Introduction

Dental caries (tooth decay) remain a significant health issue amongst preschool children worldwide [1]. The last National Dental Epidemiology Programmes survey of five year old children in England in 2022 showed that 29.3% of children had dental caries, and on average, they had at least three untreated teeth with dental caries [2]. Dental caries negatively impact the oral health-related quality of life of preschool children and their parents [3], school performance and school attendance [4]. The cost of dental caries treatment poses a large economic burden on the healthcare system [5].

Childhood is a crucial opportunity to acquire knowledge, skills and behaviours that affect health [6, 7]. The preschool years mark a transition from dependence to greater autonomy, during which children develop physical and cognitive abilities essential for self-care, such as tooth brushing. Therefore it is an important time to establish good dental habits. It is recommended that regular tooth brushing should be commenced from the eruption of the first tooth [8]. It is recognised that dietary and oral hygiene behaviours and other health patterns become firmly established during preschool age, and any unhelpful dental behaviours (such as having fizzy drinks in the bottle or using dummies) can be challenging to alter as the child develops [9]. This is supported by a growing body of evidence that emphasizes the significance of oral health in childhood [5, 10–14] as early caries increase the risk of ongoing dental issues later in life [15]. Notably, a randomized clinical trial (with 5-year follow-up) demonstrated that early intervention for childhood caries is more effective when initiated during infancy [16]. Therefore, oral health behaviours, such as regular tooth brushing and reducing sugar intake, should be encouraged from an early age to prevent caries and promote lifelong dental health [17].

Parents report difficulty accessing dental services for their children [18], and the COVID-19 pandemic exacerbated the issue. In March 2020, dental services were highly disrupted and limited to urgent care [19, 20]. An estimated 19 million fewer treatments were delivered in 2020 compared to the same period in 2019 in England [21]. Further, a dramatic decline (98%) was reported in dental services used by children during lockdown [22]. Moreover, the number of children referred for dental general anaesthesia in Northwest England significantly declined during the lockdown period (April-June) 2020, compared to the same period the year before [23]. There is still no resolution and children and parents still experience difficulty getting dentistry appointments [24]. Hence alternative additional routes to preventative oral health care are urgently needed.

In the context of difficulty in accessing timely dental care, it is likely that children will not only miss routine check-ups but also teachable moments for promoting healthy oral behaviours. Teachable moments are opportunities to motivate individuals to adopt risk-reducing health behaviours spontaneously [25]. It has been argued that routine dental check-ups in the NHS offer these opportunities [26]. McBride and colleagues identified three elements that make the teachable moments effective. These are (i) increasing the perception of personal risk and outcome expectations, (ii) redefining self-concept or social role and (iii) prompting strong effective or emotional responses [25]. The health behaviours associated with children’s oral health that are particularly relevant to discuss within dental clinics include oral hygiene practices, diet and sugar consumption, and attendance at routine appointments [26]. However, with the limited access to such services it is imperative to find teachable opportunities beyond dental clinics to provide oral health support for children and their parents.

The early year’s foundation stage (EYFS) framework sets the standards to ensure that children aged from birth to five years learn and develop well and are kept healthy and safe [27]. It has been argued that it is possible to minimise or even eradicate dental caries in children by targeting children and parents in childhood centres [28, 29]. Moreover, oral health improvement activities such as supervised toothbrushing programmes have been shown to improve oral health and reduce inequities [30, 31]. The UK government therefore consulted 2452 representatives from different positions (teachers, parents, managers, local authorities and others) to seek their views on incorporating oral health within the early years framework to promote children’s oral health [32]. The responses were mixed; some respondents agreed oral health should be included in early years settings, while others disagreed, believing that oral health care was the exclusive remit of parents and healthcare professionals [32]. There was no indication of which stakeholders (i.e. EYS versus Parents) accepted and disagreed with the idea or an EYS oral health agenda. Further, no research has systematically explored the reasons underpinning these differing views.

In response to the consultation, oral health was included within the framework, with each setting being able to determine its own requirement [32]. These changes were introduced in September 2021 [27]. However, the guidance lacks specificity on how EYS should promote oral health and it is left to the discretion of staff as to how to promote oral health within their settings. The guidance lacks details, stating simply that *‘The provider must promote the good health*,* including the oral health*,* of children attending the setting*’ [33, p.32]. There have been more recent guidelines (after the data in the current study were collected) which do provide some additional suggestions as to how oral health could be promoted. These include supervised toothbrushing programmes, dentist role play and sharing oral health resources with parents and carers [34]. However how these ideas could be implemented and support around overcoming potential challenges staff have in initiating conversations about oral health with parents, carers and children remain lacking. Hence, it is important to understand how EYS integrate oral health into their activities and how families perceive those activities.

This study aims to investigate parents’ views about the role of EYS and childcare providers in promoting oral health, whether there are any opportunities to provide behaviour change conversations at EYS, and if they could mitigate the difficulty of accessing dental services as well as parents’ perspectives about those conversations.

## Method

A qualitative study was conducted comprising individual semi-structured interviews with parents of children aged under five years who were enrolled in any early-year settings in England. Approval was received from the Ethics Committee at the University of Manchester (2022-13824-24563) and (2023-17885-31309). For transparency and rigor in reporting, the COREQ (Consolidated Criteria for Reporting Qualitative Studies) 32-item checklist has been completed for this study and is available as Supplementary File 1.

### Participants and recruitment

Participants were recruited through social media advertising and from the lead researcher visiting Sure Start centres and contacting local nurseries and early year settings. The research team had no previous relationships with these settings prior to embarking on the study. The eligibility criteria were adults aged 18 years or over who were caregivers of at least one child enrolled in an EYS in England. Participants needed to be fluent in English or Arabic. Recruitment took place between July to September 2022.

There are different types of EYS in England that meet the needs of children from different ages and socioeconomic groups. These include nurseries, preschools, Sure Start centres, childminders and Madrassas. At the time of recruitment, all three and four-year-olds in the UK received up to 15 h of free early education and childcare per week for 38 weeks of the year [35], with proposals to extend this in near future for working parents of children aged nine months and above [36]. 

Interested participants were given an information sheet and the opportunity to ask questions before providing written or verbal consent to participate in the study. Potential participants had at least 24 h to decide whether to take part. Demographic information included the type of early years setting their child was registered at, the settings’ postcode, number of children enrolled at an EYS, children’s age, whether children were registered with a dentist, date and reason for their last dental visit, parent’s work status, parent age, gender and ethnicity. The EYS postcode was converted to an Index of Multiple Deprivation (IMD) decile. IMD deciles toward one indicate a more deprived area, and decile ten is the least deprived [37]. The information sheet, demographic questionnaire and consent form were translated into Arabic, and participants had the option of undertaking the interview in Arabic or English. A £10 high street voucher was emailed to each participant after the interview as a thank you for their contribution.

### Data collection

The interviewer followed a topic guide that comprised of open-ended questions and prompts. The topic guide was initially generated based on the purpose of the study and a review or relevant literature. It was further developed through two stages. Firstly, through discussion with the wider research team, where further questions were added and refined. The second stage involved piloting the interview schedule with a mother of two children, of whom one was enrolled in nursery/preschool. Based on her suggestions, some questions were rephrased to improve clarity. The final guide covered five topics: (i) oral health support and activities provided by EYS for their children, (ii) perception of oral health inclusion to EYS framework, (iii) experience in receiving any oral health related promotion from EYS staff, (iv) communication and relationships with EYS staff and (v) oral behaviour change conversations with staff and implementation of related behaviours at home. (Please refer to supplementary document 2).

Interviews lasted for 18 to 79 min (mean = 38). All participants who expressed an interest in the study took part. Fourteen parents were interviewed. Eight interviews were conducted in Arabic, and six in English. One interview was conducted face-to-face (in a quiet room on a university campus). The rest were conducted online using the video conferencing software Zoom. No other adults were present aside from the interviewer and parent during the interviews. Online interviews have been identified as having the advantage of widening access to participants, time- and cost-efficiencies [38], and allowing participants to fit the interviews into their busy schedules and save on travel expenses and commute time [39, 40].

Interviews were audio-recorded and transcribed verbatim. Any identifying information (e.g. names and place), were removed. All interviews were conducted by the first author, who is a bilingual female, non-parent researcher with a clinical background in dentistry, studying for a PhD. The three other members of the research team were also female. They were academic psychologists with expertise in healthcare communication, dentistry, behaviour change and qualitative methods. All were parents, and one had a child in an EYS at the time of data collection. Following interviews and throughout the recruitment process, the first author kept field notes of her reflections.

Arabic interviews were transcribed and analysed in the original language, with quotes then were translated into English using a stepwise approach. By analysing data in the same language it was collected in, the meaning of the data can be preserved during analysis [41, 42] and the accuracy of the translation can be checked more efficiently using small chunks of text. The first author translated all the quotes into English to maximise the translation reliability [43, 44]. In the second step, two bilingual translators independently assessed the translated quotes using previously developed criteria and suggested any changes. All three translators met to discuss each transcript and agree on the final translation. Subsequently, a further bilingual individual translated the English version back to Arabic and the original translators compared this with the original Arabic quotations to ensure meaning was not lost [45]. Detailed information about the translation approach can be found in a separate publication [46].

### Data analysis

Analysis was inductive, and followed reflexive thematic analysis approach (RTA) by Braun and Clarke [47]. In phase one, interviews were read repeatedly, allowing the lead researcher to become familiar and engaged with the data. In phase two, a more formal coding process was conducted, which involved discussion with the research team. The scripts were organised and coded using a computer-assisted qualitative data analysis software [48], as this kind of software provides an organised storage system that can easily and quickly access the materials and export the codebook [49]. In phases three and four, themes were generated, developed, reviewed, and discussed with all contributing authors. Analysis and interpretation was at the latent level. In phase five, we further refined the developed the themes, including defining and naming them. Reporting findings were considered through all phases rather than as a discrete phase. All members were involved in this process. The final report was organised and drafted by the lead researcher and reviewed, offered feedback and edited by the other authors.

## Results

The fourteen participants comprised 11 mothers and 3 fathers. Participants had a total of 19 children (range 1–3 per participant). A summary of participant’s and children’s information is provided in Table 1.

**Table 1:** Participants’ demographics information

Parent’s information (Total = 14)	
Age range	• 23 to 39 years (mean = 29)
Region	• North-West England (*n* = 6) • Yorkshire and the Humber (5) • South-West England (1) • East Midlands (1) • London (1)
Parents’ ethnicities	• Arab (*n* = 8) • African (3) • Asian (2) • White (1).
Children information Total (19)	
Children’s age range	• 22 months to 5 years (mean = 3.3 years)
Children’s setting types	• Preschools (*n* = 7) • Nurseries (11) • Sure Start centres (1)
IMD decile for children’s settings	• 1 to 5 (more deprived) (*n* = 16) • 6–10 (least deprived) (3)
Dentist registration	• Registered (*n* = 12) • Not registered (7)

**Fig. 1:**
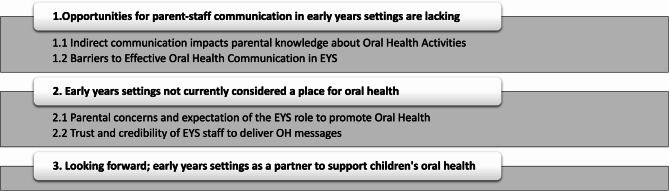
Study themes

Three themes were developed to reflect parent’s views about the role of EYS and childcare providers in promoting children’s oral health (see Fig. 1). Participants quotes are presented within the text to illustrate these themes.

### Opportunities for parent-staff communication in early years settings are lacking

This theme explores the limited communication opportunities between parents and staff in early years settings, which can impact parental knowledge and engagement with oral health practices. It is organised into two subthemes: indirect communication impacts parental knowledge about oral health activities and barriers to effective oral health communication in EYS. The first details parents’ understanding of current oral health practices and resources provided by EYS. The second then investigates the communication barriers that contribute to this uncertainty, highlighting areas for improvement.

### Indirect communication impacts parental knowledge about oral health activities

No parent was aware that oral health was part of the EYS framework. *‘No*,* no*,* no*,* not that I remember*,* P10’.* A few parents recalled EYS delivering some OH activities, however these were described as ad hoc and focused primarily on toothbrushing and providing toothbrush-related goody-bags. A minority of parents reported that their child brushed their teeth at their EYS.

Parents reported that understanding about what oral health care occurred within EYS was assumed and they were uncertain about what, if any, oral health-related activities took place. For example, a mother asked her children during the interview. Others reported that they could not recall seeing evidence of OH-related activities in the nursery, and had not heard reports of OH-related activities from their children.

*‘Frankly*,* I am not sure*,* but usually*,* I mean my daughter*,* when she knows new thing*,* when she learns new things*,* she say*,* “mum”*,* for example*,* “the school said this*,* the Ms said this.” So*,* she tell me. But*,* about this point (oral health) honestly*,* I am not sure. And always*,* when I enter the nursery*,* I see all the toys they (the children) play with. So*,* mostly*,* there are kitchen stuff. Kitchen stuff*,* building blocks*,* and stuff like this. I never happen to come across*,* for example*,* mouth model or toothbrush*,* or anything like that. I mean*,* I didn’t see this type of education’(P9).*

Parents often did not recall receiving resources and information regarding oral health *‘I don’t think I have ever get any information about that’ (P6)*. Where participants had received resources, these were provided in the form of a leaflet (paper or digital) and focused on effective toothbrushing, choosing the right toothpaste, sugar consumption, and breastfeeding. Parents considered that leaflets alone were insufficient as they needed someone to explain to them how to use and implement the information. They felt further support from EYS staff was necessary to make these useful.

*‘Then they give you with a storybook in it and all the information about brushing children’s teeth but they don’t tell you like*,* they give you a paper and showing you how to do it but they don’t do it physically with you there’ (P4)*.

### Barriers to effective oral health communication in EYS

While parents interact with staff in multiple situations (e.g. drop-off/pick-up, one-to-one meetings, inductions, parent events) most communication occurs in written form. This include parents receiving emails, a daily logbook, mobile applications, Facebook communities and websites. Email was the most used method used by staff to communicate information. Participants preferred this as it was convenient, familiar and could be referred to repeatedly if needed. Such messages could however be missed as there was a lot of email-traffic, which was described as often being irrelevant, information-dense, poorly formatted and unengaging.

They sent to you like this: everything in black AND bold. Everything black AND bold. You will not understand anything. They say this staff had relocated, we hired a new teacher, and so on. So what? The toilet water was shut off, so? You know, I do not want to know all that, (P2)

Altogether parents were overwhelmed by the information received and discouraged from sifting through it to identify what, if any, actions were required of them. In this context information relating to tooth-kind activities or actions was easily missed.

Specific barriers to accessing the information given such as leaflets included not being able to understand what was written as they had insufficient English, difficulty finding time to read them, and having to keep them away from children who might tear or draw on them. Parents also felt uncertain how to use the resources staff had given them.

*‘I believe they should have more classes and speak to parents individually*,* rather than just giving them leaflets to read by themselves because like other parents*,* they wouldn’t know how to read’ (P6)*.

### Early years settings not currently considered a place for oral health

This theme focuses on parents’ views about how suitable they considered EYS and staff is to support their children’s oral health. It is organised into two subthemes. The first focuses on parents’ views on the role of EYS’ and their appropriateness for conducting OH activities. The second describes how credible EYS staff were perceived as being in delivering oral health messages.

### Parental concerns and expectation of the EYS role to promote oral health

Parents viewed EYS as having two fundamental purposes, which influenced their views about how appropriate this setting is for conducting OH activities. Some parents viewed EYS primarily as a place of education and learning for the child, whilst others viewed it exclusively as a place of safety to care for children whilst parents were unable to. Some saw EYS as having both functions.

*‘I do not care about the extra things they do. Because for example*,* the goal is for the boy to go to the nursery is to be in a good place*,* learn and interact. Did you understand? I am not expecting anything else from them*,* if we could say’ (P2)*.

The financial cost of the EYS determined the level of service parents expected, with a belief that more expensive settings would provide a wider range of services. Parents considered oral health and its activities to be a luxury, hence more likely to be expected in pricier settings. Some parents said they expected toothbrushing to be incorporated into EYS because children spent all day at EYS and would have at least one meal there.

*‘I expect it might be the private (EYS) that has very good services*,* for example. I expect they are the ones implementing this thing (oral health activities)*,* I don’t know’ (P9)*.

*‘I expect because the girl (her daughter)*,* will have a meal*,* and there will be eating*,* and there will be a meal-time in there. I expect that they will request from me a toothbrush and toothpaste. This is my expectation*,* but it was not included (In the list they asked the parents to bring)’ (P12)*.

Most participants expressed some concern about toothbrushing at EYS, with hygiene being a key consideration. Parents worried that children might exchange their toothbrushes, queried how they would be stored, and how they could brush while they have limited sinks.

*‘schools are like clean but you never know*,* germs can be everywhere and they’re unseen’ (P4)*.

Parents also raised concerns about the safety of fluoride and its impact on their children’s health, particularly the risk of ingestion, as young children may not have access to sinks to rinse their mouths after brushing. Moreover, parents thought brushing at EYS wouldn’t provide additional benefits since their children already brush twice daily at home, and some linked it to the dental guidelines. *‘I guess the general guidelines that we’ve gotten in the UK is that you just need to brush your teeth two times a day’ (P10).* Some also believed that introducing toothbrushing to older children would be more effective as they can better understand its importance. Others highlighted the difficulty of controlling multiple children at once and questioned whether staff would have the capacity for any additional tasks. This view was aligned with parents’ experience of a lack of staff, leading to occasions when parents were asked not to bring children as the setting had to close.

While most parents were concerned about toothbrushing at EYS, a few parents highlighted how the inclusion of oral health activities positively impacts children and parents. Parents noticed that brushing at EYS encouraged their children to brush at home, and children were more likely to remind their parents to brush when parents forgot. These parents noticed that children brushed more regularly at home during term time compared to school holidays. One parent described choosing to implement the same brushing technique used at EYS at home. Further, parents found that toothbrushing reward charts provided by their children’s settings encouraged their children to brush at home and maintain the habit.

‘*she wasn’t that excited about it at first. And then all of a sudden*,* she came home and she was excited about it and she would do it. And she would do it for the proper amount of time and without us having to like really ask her to do it. So we attribute that to doing it at school and doing that with her friends’ (P10)*.

### Trust and credibility of EYS staff to deliver OH messages

Parents reported that it did not occur to them to ask EYS staff about oral health. Some explained this was because they had no prior experience of oral health-related conversations with EYS staff. They believed that the relevant professionals to discuss oral health with are a dentist or health visitor. Other sources of oral health information they had either sought or considered appropriate were online via YouTube, NHS websites, parenting websites and from other parents within their social network.

*‘I feel like I search for the information from its source*,* either I look online on the websites that talk about children and their development. Or possibly ask the health visitor about specific things. Which I did previously.’ (P7)*.

Whilst some participants felt they would only accept oral health advice from dental healthcare professionals, others did think it would be acceptable to have conversations with EYS staff about basic children’s oral health information. Parents emphasised the importance of tailoring any information (regardless who it came from) since not all parents have the same level of health literacy. Participants argued that staff should have additional training in oral health in order to undertake such activities and that going beyond their expertise was potentially harmful or could ‘c*ause chaos’ (P12).* In contrast, other participants, argued that there was no need for specialist training because everyone should know the basics of oral health.

*‘I’m not really sure about that but I think like something related to oral health*,* like a certification. I don’t know if there’s any certificate. Usually they always have some kind of like a safety certification. Maybe this one is from a dental or oral health certification’ (P6)*.

### Looking forward; early years settings as a partner to support children’s oral health

This theme explores parents’ perspectives on the potential role of early years settings (EYS) as supportive partners in promoting children’s oral health. It highlights the qualities and characteristics that parents believe are essential for EYS to effectively fulfil this role. Although all participants believed that their children’s oral health was primarily a parent’s responsibility, several felt that EYS staff could share this role with them. There were several reasons why staff were valued for their input. Parents argued that children spend most of their day (and at least one meal) at the EYS, that staff have the required skills to convince children to do any tasks they ask them to do (e, g, toothbrushing) and that children tend to follow their teachers’ instructions more than their parents’.

*The child is encouraged by the teacher because the teacher helps the child and encourages him even more than the mother’ (P13)*.

Moreover, parents argued that there was added benefit to working as a parent-educator team and that having a consistent approach helped children generalise healthy behaviours across the settings.

*‘When there is a cooperation from the parents*,* and an acceptance from EYS*,* or if they incorporate it in their curriculum. The child will have the mindset to do it (toothbrushing/ oral care). I have to do it because it is a must. Regardless of the place I’m at. Either I was home or at school’ (P12)*.

Parents believed that children would benefit from incorporating oral health into the EYS framework for many reasons. First, it would provide children with essential knowledge regarding oral health, particularly for children of caregivers with less knowledge. Second, children are curious and want to know why they must do certain things, such as brushing their teeth. Third, by including oral health in the EYS framework, parents believed that children would learn the importance of oral health if they heard the same information from two sources, i.e. their caregiver and their teacher.

*‘The EYS*,* by incorporating this (oral health) in the curriculum*,* is considered a positive step as it will satisfy the child’s curiosity. It will answer their whys and provide the children with the required information that they need. So they will have believe from the inside to do this (oral care) as a part of their health. (P8)’.*

Some participants believed toothbrushing at EYS would make a positive difference. Brushing with their peers help encourage children to brush. Also, children tend to impress their teachers, and the praise received from their teachers would encourage them to brush.

*‘Because the child’s nature loves to imitate his friends*,* when the teacher asks them to imitate each other in brushing their teeth*,* they will take it as an approach for the rest of their lives. (P13)*

In contrast, a small minority of the participants believed that staff at EYS should not have any responsibility for their children’s oral health. This was because they linked the responsibility for oral health care with clinical examination and treatment, and hence the sole remit of a dental practitioner. Moreover, they believed giving this responsibility to EYS staff beyond their capacity and would unfairly place a burden on them if oral health issues occurred. *‘to say that they are the responsible person is a bit too heavy’ (P6)*.

## Discussion

Parents were unaware that oral health now forms part of EYS framework and were only conscious of a very limited provision of oral health activities taking place within the setting. What awareness they had come largely via their children rather than directly from care setting staff. Hygiene and safety were key concerns parents had about conducting supervised toothbrushing at EYS. Some parents did not consider approaching EYS staff for support with their children’s oral health, did not view EYS staff as being credible or qualified, and were reluctant to accept oral health advice from a non-dental healthcare professional. However, there were others who believed that EYS staff could share responsibility for supporting their children’s oral health since the children spent most of their day in their care. Parents whose children’s setting did provide some oral health activities reported a positive impact on them and their children. They viewed this as a valuable addition to the service provided, which, if implemented, could be generalized across settings and confer lifelong habits and health benefits to children.

Communication between parents and EYS staff was reported to be both verbal and written. However, the health education information provided was often unengaging and frequently overlooked. Although EYS primarily communicated with parents via emails, the number of emails and their contents and formatting discouraged parents from reading them. The use of digital tools for communication, such as emails, has many advantages, as they are efficient, immediate, and offer equal access to parents [50]. However, it remains often a one-way transfer of information and may not address the variation in the linguistic competence of parents. Moreover, it is guided by staff intentions rather than parents’ needs, expectations and intentions [50]. These findings suggest that staff need to support on how to communicate more effectively using digital communication and tailor it to parents’ needs.

Indirect communication via children was considered a barrier to parent communication in educational settings [51]. Successful implementation of healthy lifestyle interventions requires two-way communication between home and school [52]. The child is at the centre of communication between parents and staff in childcare settings [53]. Indeed, parents in our study relied on their children to know about any oral health care and education that was occurring. Children were partly filling the gap in parent and staff problematic communication. Hence, any communication about the child can be considered triadic communication, involving multiple dyadic interactions between the different parties, which has only previously been examined within dental settings [54]. In school settings it is likely to be inadequate and fail to address the concerns that parents have. An interactive intervention that engages children, parents, and staff may address this gap.

Parents expect EYS to be a place of education and/or caregiving. These beliefs played an important role in how acceptable they found staff involvement in supporting their children’s oral health. Henderson and Rubin, identified two key barriers to involving parents in oral health activities in EYS: the nature of the relationship between staff and parents, and the fact that parents do not prioritise oral health [55]. The findings of this study support this, as parents demonstrated limited awareness of oral health initiatives in EYS and expressed uncertainty about staff roles in oral health promotion. Additionally, some parents deprioritised oral health due to competing demands, time constraints, and a lack of clear communication from staff about the importance of these activities. This highlights the need for EYS staff to actively engage parents, ensuring that oral health promotion is positioned as an integral part of children’s overall well-being.

The lack of parental involvement and awareness can also be attributed to limited communication between EYS and families. This was evident, as none of the parents were aware that oral health had been incorporated as a mandatory component in EYS. Therefore, EYS staff need to improve parental engagement and establish stronger partnerships. Education Scotland developed a comprehensive toolkit that guides EYS staff to parental involvement and engagement in their settings [56]. The toolkit consisted of 11 sections and includes topics such as the benefits of involving parents in the children’s learning, home and school partnerships, and parental representation, with ideas about activities that can done with parent groups. Yet, the toolkit lacks clarity on how to navigate and utilize the various sections, as many topics overlap. Providing a more structured guide on how to approach the toolkit could enhance its usability for users.

Some parents in the current study reported they were willing to receive oral health messages from the staff. It is important for parents to be approached in a non-judgmental manner. Parents accept healthy advice from staff in their children’s settings if it is not perceived as being told what to do [57]. This could also explain why some parents may opt to seek information online. It has been emphasised that staff need to build up a relationship with parents before promoting health, and that they can engage parents in conversations by ensuring that their approach is non-threatening, approachable, and not preachy [55]. So, it appears that staff need a variety of skills to engage parents in meaningful oral health discussions without damaging the trusting relationship needed in a caregiving setting.

Hygiene and fluoride safety were two concerns that parents had against implementing supervised toothbrushing at EYS. Parent concerns regarding hygiene might have been exacerbated by COVID-19 pandemic and differentially worry different groups of parents. In a study by Woodland et al., parents who lived in the North of England and those from black, Asian and ethnic minority backgrounds were least likely to send their children back to their education settings when they reopened after the lockdown [58]. This might be reflected in the parents in this study as they were mostly from these groups.

Parents’ safety concerns about supervised toothbrushing in early years settings could be alleviated by increasing their awareness of the existing protocols and ensuring these are effectively communicated [59]. Regular monitoring by oral health improvement teams, commissioned by local authorities, could further reassure parents about the program’s safety and implementation. Also, the parent could be informed of the dry toothbrushing technique, which do not require use of sinks [60]. Safety concerns also reflect that parents need to know about the correct behaviours for toothbrushing, that there is no need to rinse the toothpaste (children should spit out the excess) in order to maximise benefit from the fluoride [60]. Furthermore, those concerned about fluoride safety need additional support to raise awareness [61].

### Study limitations and strength

This is the first study to take an in-depth systematic approach to investigate the views of parents of EYS practice around oral health education. The findings are timely and important evidence to support the existing rollout and proposals for expanding oral health practice at EYS nationwide.

Our study covered a diverse group in terms of ethnicity, area of deprivation, and geography. The data were collected across a range of settings and locations, in two languages (English and Arabic), and in two different methods (in person and virtual). Hence, we enabled the integration of several underrepresented groups into our study [62] which is important to ensure that research findings are relevant, reproducible, and address the needs of different communities [63]. Yet, it is important to recognise that it is likely our sample likely includes individuals who are interested in the topic of child oral health and hence may overestimate parents’ knowledge of and willingness to engage in oral health conversations with EYS staff. Whilst parents willingly talked about their concerns, the data corpus may not capture the full range of these.

Most participants were mothers. Efforts were taken to include fathers, but only three took part. Mothers are still often primary carers and the person in the household who takes on responsibility for oral health care. Mothers report a conflict with their partners about their children’s oral hygiene and diets and consider them as more lenient [64]. Fathers’ lack of participation in research is an issue for the broader field and it was important we could, to some extent, include their voices which are often overlooked [65, 66].

### Implication and future direction

While parents reported limited awareness of oral health activities being undertaken by early years settings, it is possible that a greater variety of practices exists than parents are aware of. Future research is needed to explore these practices in more depth by engaging directly with EYS staff. This highlights a dual issue: variability in the implementation of oral health activities within early years settings and the extent to which parents are informed about these efforts.

The findings reveal the need to intervene to increase the credibility of staff to parents in promoting children’s oral health within early year settings. Additional training and support for staff is needed as to how to communicate more effectively with parents about oral health care in ways that are engaging and acceptable. Further work is needed to investigate other stakeholders, in particular hear the views of EYS staff themselves, as they are key actors in implementing oral health education and care.

## Conclusion

This study contributes to the limited evidence base by providing a clearer understanding of parents’ views on the current practice of oral health activities and communication to promote children’s oral health at EYS. Parents reported limited awareness of oral health activities in the EYS curriculum and multiple barriers are operating that prevent useful oral health-related conversations from occurring. Although parents believe that children’s oral health is primarily their responsibility, there is recognition of a potential role for EYS staff involvement in supporting child oral health behaviour change. To achieve this will involve resources and support for EYS staff on how to communicate with parents regarding their children’s oral health in order to reduce and prevent harmful dental diseases.

## Electronic supplementary material

Below is the link to the electronic supplementary material.

Supplementary Material 1

Supplementary Material 2

## Data Availability

The data that support the findings of this study are available from the corresponding author upon reasonable request.

## Declarations

### Declaration of Helsinki

The research adhered to the declaration of Helsinki.

## Abbreviations

EYS: Early Years Settings
OH: Oral Health
