# Supplementary material for: Caregivers’ views on childcare staff’s role in promoting children’s oral health
Source: BMC Oral Health. 2025 May 24;25:790. doi: 10.1186/s12903-025-05946-y (PMC12103805; doi:10.1186/s12903-025-05946-y)
Supplement: Supplementary file 2 — Supplementary Material 2 [file 12903_2025_5946_MOESM2_ESM.docx]

**Supplementary document (1)**

**Interview guide**

| Aspect addressed | Interview questions |
| --- | --- |
| Current practice of supporting oral health for children's in preschool settings. | 1. Does your preschool/ childminder provide any activities to support/ education children about oral health?   If yes, what kind of activities are you aware of?   1. Has (your child/ child under your care) ever had their teeth brushed or brushed their teeth at preschool or with their childminder?  - Can you describe what happens? - Who provides the toothbrush/ toothpaste - How do you know? [Probe for how information is communicated]  1. Are there any resources that preschools or childminders share with you or your child regarding's children's oral health?  - E.g. leaflets, apps, websites, free toothbrushes |
| Caregivers' views and perceptions about including oral health in preschool curriculum | 1. Do you think oral health/supervised tooth brushing is part of the preschool/early years foundation? 2. What are your thoughts on involving oral health in the preschool curriculum?  - Do you think preschool is an appropriate place for tooth brushing? - How do you think preschools should support oral health?  1. Do you think that preschools staff are responsible for supporting your children's oral health?  - If **yes**, why/ how? If **no**, Why not and who is responsible? |
| Experiences of receiving any oral health related promotion form the childcare's providers. | 1. Have you ever had information related to children's oral health from their preschool?    - What was it?    - What did you think if it? 2. Have you ever contacted preschool staff with concerns about your child/children’s oral health?  - What was the response? - Were you satisfied with the response? |
| Exploring the rapport between preschool staff and caregivers | 1. What kind of communication do you have with preschools about you children?  - Notes, emails, mobile applications, websites, forums  1. What are the opportunities for one-to-one conversations about children between you and the preschool staff?  - Children's health, any issues and concerns you have, educational progress - Formal opportunities e.g. parents evening - Informal opportunities e.g. drop off and pick ups |
| Caregiver's acceptance and expectation of receiving oral health conversation from preschool staff in the future. | 1. How would you feel if preschool staff talked to you about how to help your children to look after their teeth ?  - Tooth brushing - Reducing sugar consumption - Attending dental appointments  1. What sort of training do you think they would need first? 2. How would you like to receive support/ advice with your child/ren’s oral health?  - F2F, written note, online, text |
